# Drug‐releasing intravesical floating technology for sequential gemcitabine and docetaxel in non‐muscle‐invasive bladder cancer

**DOI:** 10.1111/bju.70060

**Published:** 2025-11-03

**Authors:** Ashley C. Rhodes, Kaitlyn A. McClintic, Emily Witt, Ikenna Nwosu, Kyle R. Balk, Colin Reis, Ian C. Sutton, Jianling Bi, Melinda Z. Fu, Michael A. O’Donnell, Vignesh T. Packiam, James D. Byrne

**Affiliations:** ^1^ Department of Biomedical Engineering Duke University Durham NC USA; ^2^ Department of Biomedical Engineering University of Iowa Iowa City IA USA; ^3^ Department of Radiation Oncology University of Iowa Iowa City IA USA; ^4^ Department of Urology University of Iowa Iowa City IA USA; ^5^ Carver College of Medicine University of Iowa Iowa City IA USA; ^6^ Holden Comprehensive Cancer Center University of Iowa Iowa City IA USA; ^7^ Division of Urologic Oncology, Department of Surgical Oncology Rutgers Cancer Institute New Brunswick NJ USA; ^8^ Women and Children's Hospital of Chongqing Medical University Chongqing China; ^9^ Chongqing Health Center for Women and Children Chongqing China

**Keywords:** non‐muscle‐invasive bladder cancer, gemcitabine, docetaxel, drug delivery, intravesical, chemotherapy

## Abstract

**Objectives:**

To develop a drug‐releasing intravesical floating technology (DRIFT) device for controlled sequential delivery of gemcitabine and docetaxel (Gem/Doce) to optimise the treatment of non‐muscle‐invasive bladder cancer (NMIBC) while enabling patient mobility and self‐removal, as sequential intravesical Gem/Doce has been increasingly utilised but has logistical limitations requiring prolonged clinic visits and patient immobilisation.

**Materials and Methods:**

The DRIFT device features a three‐dimensional printed perforated tube with latex sleeve, dissolvable polyvinyl acetate and polyvinylpyrrolidone end cap with adjustable fluorinated polymer (FluoroPel) coating, and patient‐removal suture. A 14‐F catheter is placed and intravesical gemcitabine is instilled. The deflated DRIFT device is inserted via catheter and inflated with docetaxel and air. The catheter is removed, allowing gemcitabine to dwell temporarily and be voided by the patient. The DRIFT device remains in the bladder and subsequently releases docetaxel in a controlled, delayed fashion, followed by patient removal. Its flexible, buoyant design supports patient mobility and maintains unimpeded urinary flow. Dissolution kinetics were evaluated using methylene blue, device performance was assessed in Merino sheep, and docetaxel tissue penetration was evaluated in rabbit bladder tissue using high‐performance liquid chromatography analysis.

**Results:**

The DRIFT device demonstrated adaptable drug release through FluoroPel coating optimisation, with dissolution times extending significantly from zero to three coatings (*P* < 0.001). Docetaxel release kinetics plateaued between 2.0 and 3.0 mL volumes. Sheep studies revealed similar timed drug release as *in vitro* testing. Escalating gemcitabine concentrations enhanced docetaxel tissue penetration, with peak concentrations reaching 0.45 vs 0.08 mg/mL in controls. Extended gemcitabine dwell time (up to 4 h) further improved docetaxel delivery, achieving significant enhancement in deep tissue penetration (*P* < 0.001).

**Conclusion:**

The DRIFT enables controlled sequential delivery of Gem/Doce, reliably maintaining docetaxel containment for up to 120 min during gemcitabine pre‐treatment. Future *in vivo* validation will establish safety and therapeutic potential. This platform has broader applications beyond NMIBC, including urinary tract infections and interstitial cystitis.

**Abbreviations**
3D: three‐dimensional
DRIFT: drug‐releasing intravesical floating technology
Gem/Doce: gemcitabine and docetaxel
NMIBC: non‐muscle‐invasive bladder cancer
PVA: polyvinyl acetate
PVP: polyvinylpyrrolidone

## Introduction

Bladder cancer is the sixth most common cancer in the United States, affecting nearly 80 000 individuals each year. Non‐muscle‐invasive bladder cancer (NMIBC) comprises the majority of these cases, representing nearly 75% of all new bladder cancer diagnoses. Standard management of NMIBC typically involves transurethral resection of the tumour followed by adjuvant BCG therapy. However, recent global shortages have prompted the need for alternative effective therapies [1].

A promising and widely utilised regimen for NMIBC treatment has emerged involving sequential administration of two chemotherapy agents, gemcitabine and docetaxel (Gem/Doce) [2, 3, 4, 5, 6, 7, 8, 9, 10, 11, 12, 13]. This sequential intravesical treatment has demonstrated efficacy in various risk strata of NMIBC, including patients with low‐grade NMIBC, high‐grade treatment‐naïve NMIBC, and high‐grade BCG exposed or unresponsive NMIBC [9, 14, 15]. The regimen is currently undergoing prospective evaluation with the BRIDGE trial (ClinicalTrials.gov identifier: NCT05538663), with the potential to be a new first‐line standard of care in most patients with NMIBC [16]. Despite these encouraging outcomes and outlook, patient adherence to the intensive treatment schedule remains a significant challenge [17]. The current administration method requires patients to spend up to 4 h per session at outpatient clinics with limited mobility during treatment, which can result in reduced compliance [13].

To overcome these logistical challenges and enhance patient convenience, we have developed a novel patient‐removable device termed the drug‐releasing intravesical floating technology (DRIFT).

## Materials and Methods

### The DRIFT Device Design

**Fig. 1:**
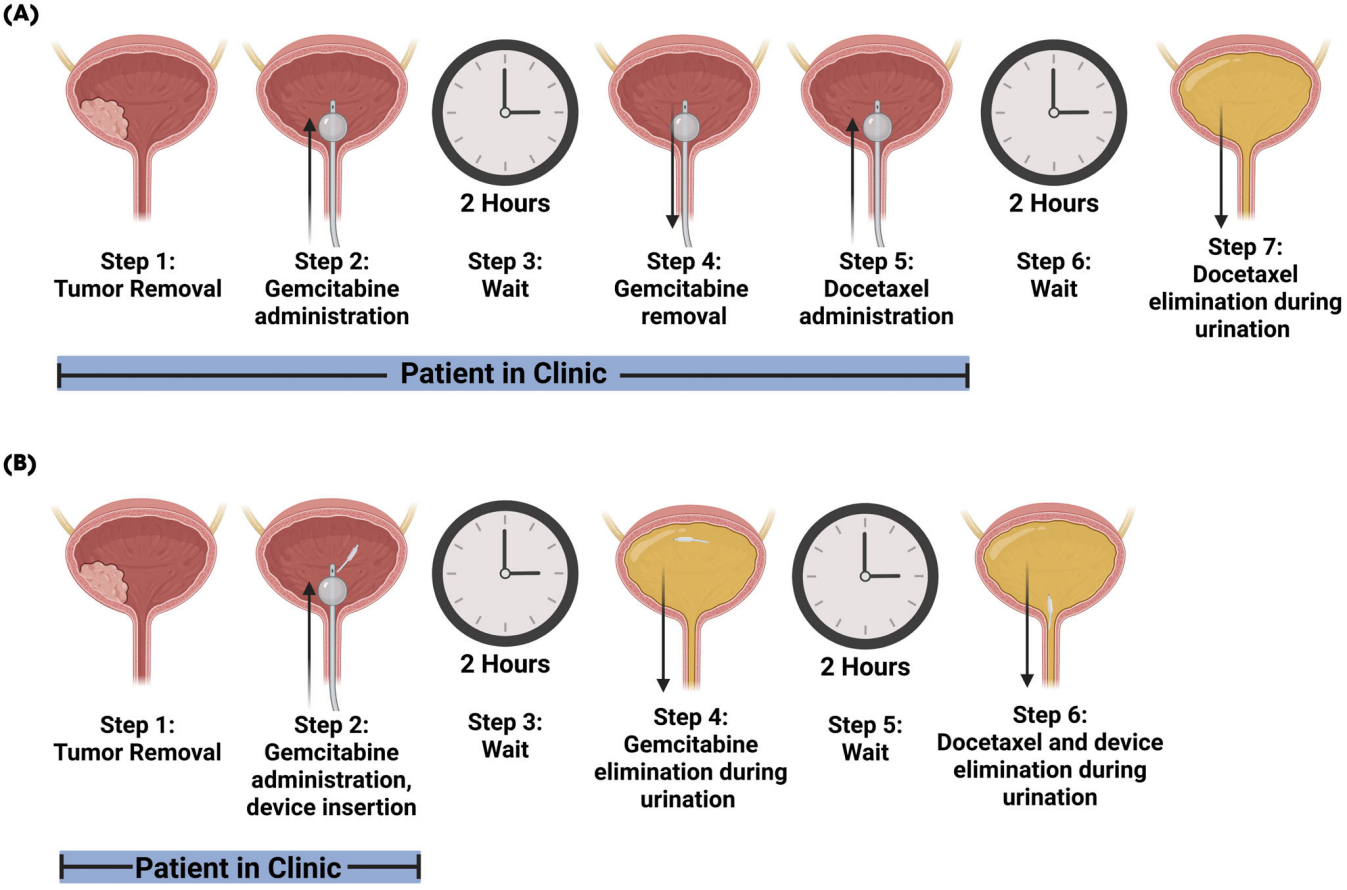
Sequential Gem/Doce regimen using the standard of care administration (**A**) compared to our DRIFT device (**B**). Created in BioRender. Byrne, J. (2025) https://BioRender.com/8cuqdxd.

The DRIFT device is designed for placement through an intravesical catheter simultaneously with gemcitabine. The device enables controlled, timed release of docetaxel and is designed to be removed by the patient, eliminating the need for cystoscopic removal. Crucially, the simultaneous administration of docetaxel via the DRIFT and gemcitabine significantly reduces the duration of outpatient clinic visits and significantly optimises logistics (Fig. 1).

### The DRIFT Device Fabrication

The internal tubing for the DRIFT device was designed using Creo Parametric (PTC Inc., Needham, MA, USA) and printed with a Formlabs Form3 three‐dimensional (3D) printer (Formlabs Inc., Somerville, MA, USA). Tubing was designed with an outer diameter of 2 mm and inner diameter of 5 mm. The design was printed using Flex80A resin (Formlabs). Balloons were created using liquid latex (Holden's Latex, Macungie, PA, USA). An inverse mould was created using 2 mm diameter tubing. The tubing was dipped twice and allowed to dry for 1 h between layers. Latex was inflated with compressed air (GUST, Reliance Worldwide Corp. Ltd, Atlanta, GA, USA) after it was dried. The latex was coated in talc (Topco Associates LLC, Itasca, IL, USA) and secured to the internal tubing using UV‐cure adhesive (Loctite, Rocky Hill, CT, USA).

Top end caps were formulated using a combination of polyvinyl acetate (PVA) and polyvinylpyrrolidone (PVP) (Sigma Aldrich, Saint Louis, MO, USA). In a Falcon tube, 270 mg PVA and 30 mg PVP were combined with 600 μL citrate buffer (pH 3) and 400 μL Eudragit EPO (Evonik Industries AG, Essen, North Rhine‐Westphalia, Germany). The mixture was stirred and applied to the end of the 2 mm diameter tubing. After drying, the end of the tubing was coated with a FluoroPel 20% dip coating (Cytonix LLC, Silver Spring, MD, USA). The other end of the tubing was filled with silicone (Sigma Aldrich) and secured with UV‐cure adhesive (Loctite). The 3‐0 polypropylene (Prolene®, Ethicon Inc., Somerville, NJ, USA) suture string is secured to the silicone end of the device through a surgeon's knot, enabling patient‐removal.

### The DRIFT Evaluation

To analyse the dissolution profile, 2 mg methylene blue was added to the PVA/PVP cap formulation, and five samples were placed into deionised water. The time until complete dissolution of the end caps was recorded. Samples of the deionised water were taken at 20‐min intervals, and absorbance was measured on a microplate reader (BioTek Synergy HT; BioTek Instruments, Inc., Winooski, VT, USA). To test the dissolution profile of the assembled device, methylene blue dye was used as a drug surrogate. The system was threaded through a 14‐F catheter and into a simulated bladder. The device was filled through the silicone end cap by a pre‐inserted 26‐G needle connected to a ~1.19 mm (3/64 inch) polypropylene tube (Fig. S1). Methylene blue dye (2.8 mL, 0.5 w/v %) was administered, consistent with the volume of docetaxel used in the clinic. Following this, 1 mL air was inserted to allow the balloon to inflate and float inside the bladder. Once the system was filled, the needle was extracted, the catheter removed, and the drug delivery system remained in the bladder. Serial images were taken as the end caps dissolved, and the dye was released into the bladder. To determine the pressure required to inflate the device, a three‐way tubing system was assembled, connecting a barometer, a 22‐G needle that was inserted into the catheter, and a 5 mL syringe. Volumes ranging from 1.5 to 3.0 mL were used to inflate the device, and maximum pressures were recorded across three devices at each volume. Background pressure required for airflow through the system without needle insertion into a device was also recorded and subtracted from the maximum pressure readings.

### Animal Studies

Male and female Merino sheep, aged 2 years, were humanely killed prior to the investigation. For ease of investigation, the DRIFT device was implanted into the bladder through a laparotomy and a small surgical incision in the bladder. The device was filled with 2.8 mL ISOVUE® (Bracco Diagnostics Inc., Princeton, NJ, USA) and 1 mL air and then deployed into the bladder. Serial X‐rays were obtained at 15‐min intervals until the cap dissolved and ISOVUE was released.

### Docetaxel Diffusion and Tissue Penetration

Rabbit bladders were obtained from the University of Iowa Veterinary Clinic and stored at −80 °C until use. Tissues were thawed, the serosal lining was removed, and samples were cut into 3.81‐cm (1.5‐inch) diameter sections. For both diffusion and tissue penetration studies, samples were incubated at 37 °C and pre‐treated with 20 mg/mL gemcitabine (MedChemExpress LLC, Monmouth Junction, NJ, USA) in artificial urine (Aldon Corp., Avon, NY, USA) for 0, 1, or 2 h.

For docetaxel diffusion studies, five tissues samples were mounted into Franz diffusion cells with PBS in the receptor compartment. Following gemcitabine treatment, 0.75 mg/mL docetaxel (MedChemExpress) in artificial urine was applied to the donor side for 2 h. Then, 1‐mL aliquots were collected from the receiver compartment every 20 min, placed in Eppendorf tubes, and stored at −80 °C until HPLC analysis. For tissue penetration studies, tissue samples (eight–15) underwent the same treatment protocol. After the 2‐h docetaxel exposure, tissues were flash frozen in liquid nitrogen and stored at −80 °C. Each sample was cryosectioned into 50‐μm slices, and 200 μg of the tissue was weighed and extracted with five times its mass of ethyl acetate (Macron). Samples were vortexed, sonicated for 20 min, centrifuged at 17 500 ***g*** for 10 min, and the supernatant was collected for HPLC analysis.

### The HPLC Analysis

The docetaxel content in the aqueous samples from both experiments was determined using HPLC analysis. The system comprised an Agilent HPLC workstation (Agilent Corp., Santa Clara, CA, USA) with an RP‐C18 column (5 μm, 4.6 mm × 150 mm; Agilent Corp.). The mobile phase consisted of acetonitrile and water (1:1, v/v), with a flow rate of 1 mL/min. The detection wavelength was set to 230 nm. Paclitaxel (MedChemExpress) was utilised as an internal standard.

## Results

### Design and Fabrication of the DRIFT

**Fig. 2:**
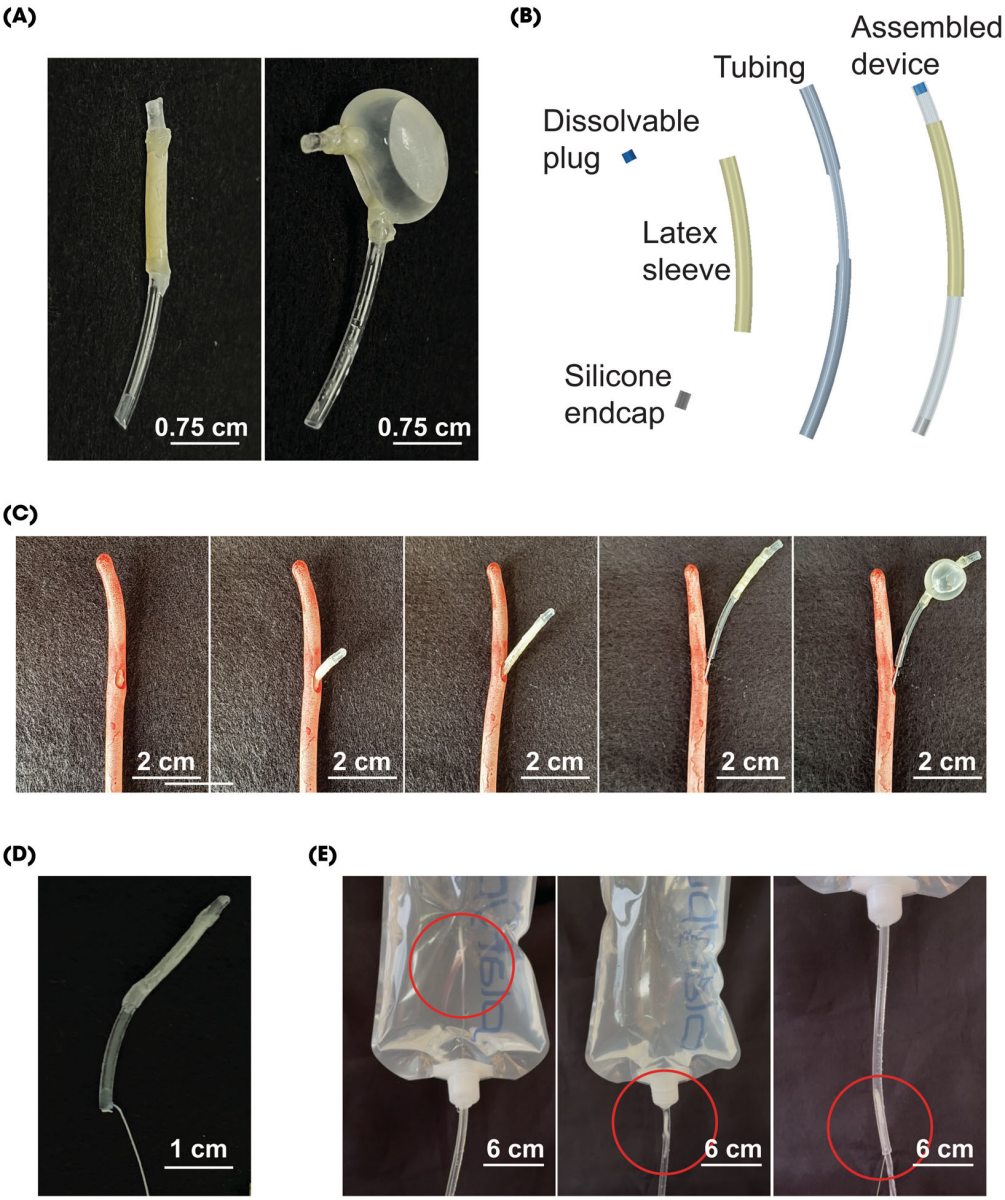
Design of a flexible, buoyant DRIFT device for the timed intravesical delivery of docetaxel. (**A**) The empty (left) and filled (right) devices have a small outer diameter. (**B**) Device components and assemblies. (**C**) The device is deployed through a 14‐F catheter and filled with the drug and air (for buoyancy). (**D**) The DRIFT device with a 3‐0 polypropylene suture attached for removal from the bladder. (**E**) Removal of the DRIFT device from a bladder surrogate demonstrating navigation through a 14‐F inner diameter tube.

The DRIFT device was specifically engineered to float within the bladder (Fig. S2) and enable the timed release of docetaxel following an initial exposure to gemcitabine (Fig. 2A). Its design features a 3D‐printed tube with multiple perforations, encased in a custom latex sleeve secured with UV‐cured adhesive. The device was capped with a dissolvable PVA/PVP end cap coated in FluoroPel and a silicone end cap for added functionality (Fig. 2B).

To insert the device, it was first deflated and passed through a standard 14‐F catheter. Once positioned in the bladder, the device is inflated with a combination of docetaxel and air using a secondary thin catheter equipped with a shortened 26‐G needle stylet (Fig. 2C). The needle stylet is removed after the device is filled. Designed to float freely within the bladder, the device minimises the risk of urethral obstruction. After the drug is released, the DRIFT device is removed by the patient by pulling the 3‐0 polypropylene suture, ensuring a seamless and patient‐friendly process.

### Adaptable Dissolution of the End Caps Allows for Controlled Release of Drug

**Fig. 3:**
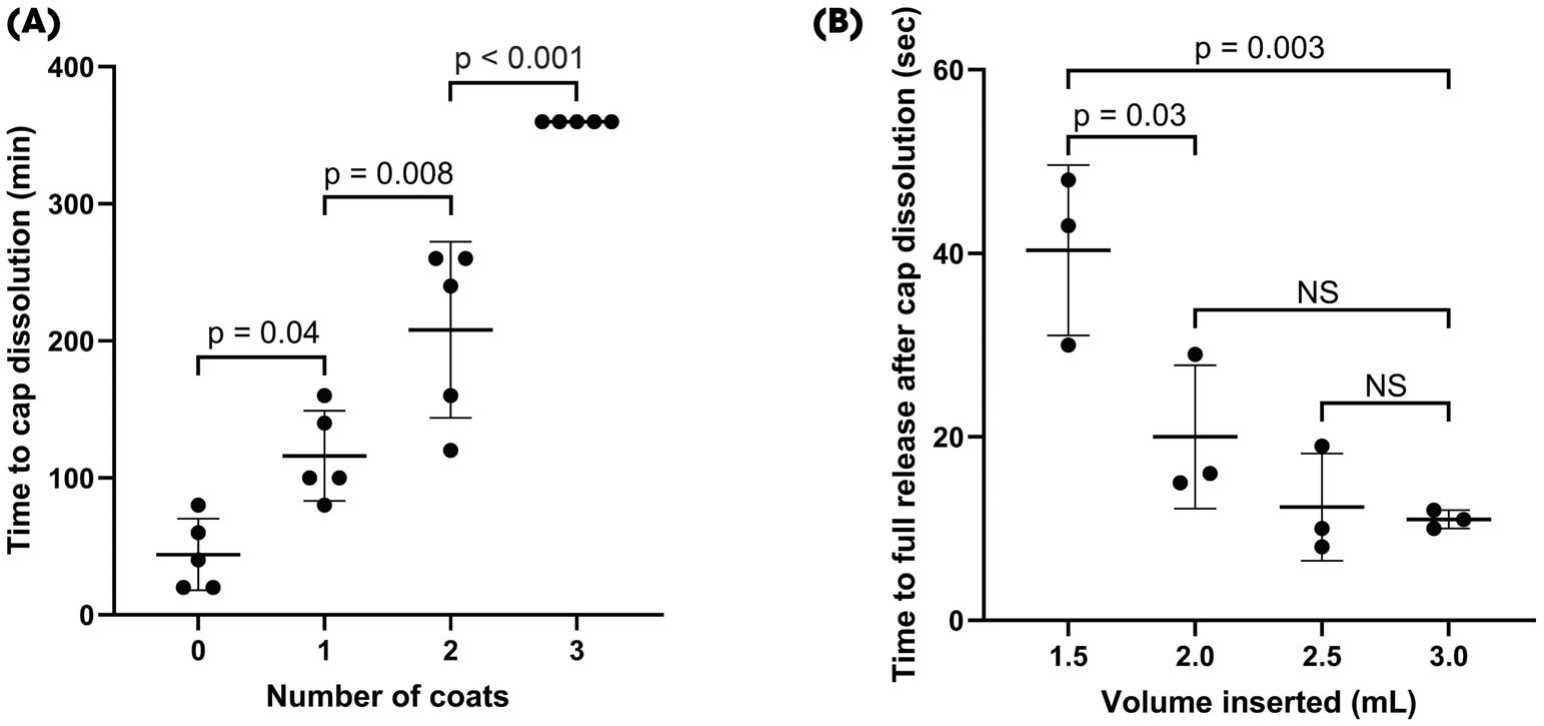
Device parameters were evaluated for the delivery of the drug. (**A**) Time to PVA/PVP cap dissolution as a function of the number of FluoroPel coats on the PVA/PVP cap. (**B**) Time to full release of docetaxel as a function of volume inserted (0.5 mL air was inserted into each device). *P* values were determined by one‐way anova with multiple comparisons. NS, not significant.

It was determined that the fluorinated polymer coating enhanced the stability and controlled dissolution timing of the device end caps. Specifically, the dissolution time of the end caps significantly increased with additional FluoroPel coatings, demonstrating an adaptable delayed release profile (Fig. 3A). Increasing the coating layers from zero to three significantly extended the dissolution duration, with the three‐coat configuration showing the longest delay (*P* < 0.001). The device's drug‐release kinetics were evaluated by varying the inserted volume of methylene blue dye solution, used here as a drug analogue. The time required for full dye release after cap dissolution significantly decreased when increasing the inserted volume from 1.5 to 2.0 mL (*P* = 0.03) and further to 3.0 mL (*P* = 0.003), indicating rapid dispersion and release capability at higher volumes. Volumes between 2.0 and 3.0 mL showed no significant differences, suggesting a plateau in release kinetics within this range (Fig. 3B). Further, the pressure required to inflate the balloon through a needle increased with volume of injectate (Fig. S3).

### Intravesical Devices Delivered Drug in Sheep

**Fig. 4:**
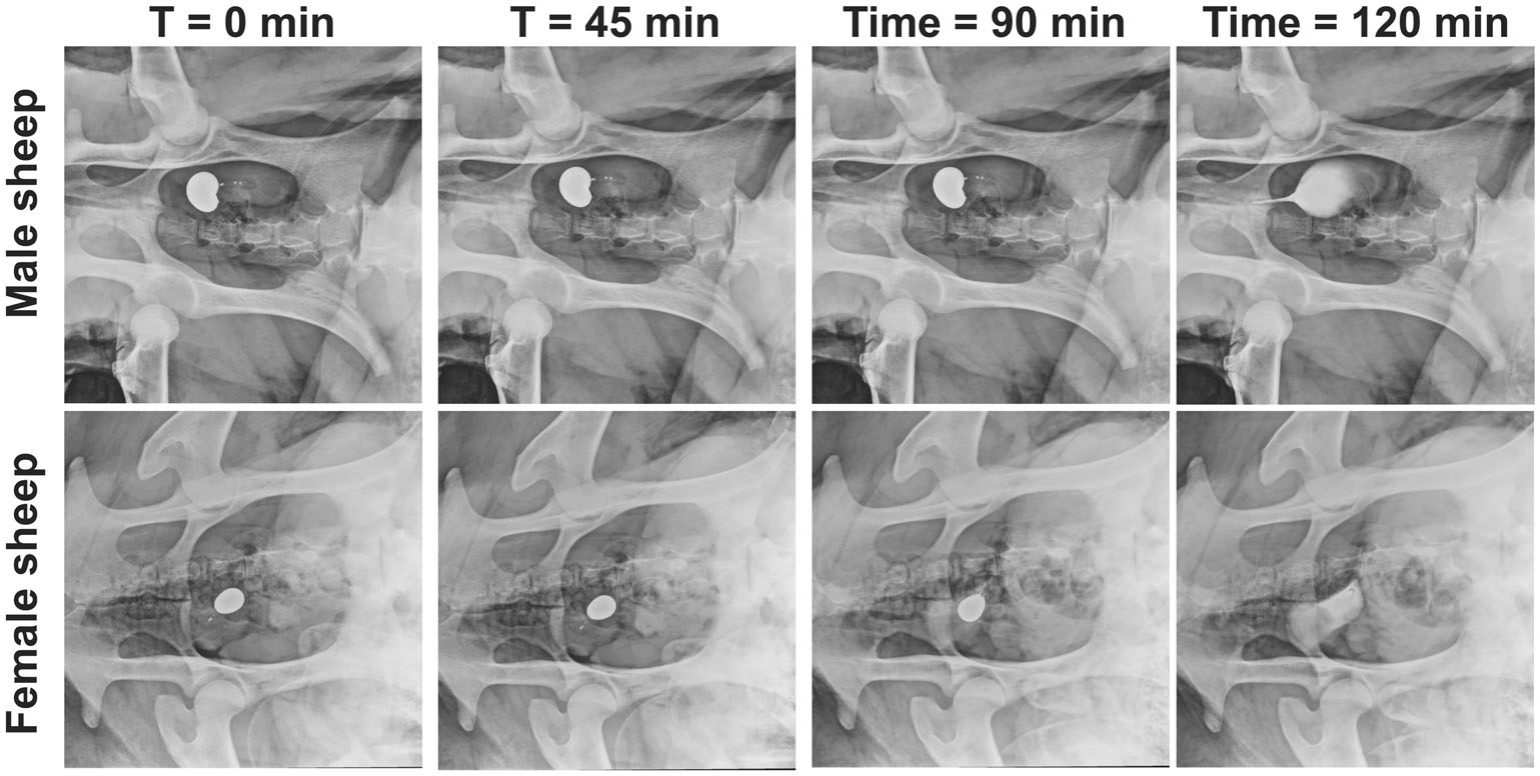
Evaluation of the DRIFT device *in vivo* in male and female Merino sheep as a function of time. The device successfully delivered a drug surrogate (ISOVUE) into the bladder at predefined times (up to 120 min). This time was chosen based on the initial Gem/Doce regimen but can be adjusted based on cap thickness.

In male and female Merino sheep, the device was deployed into the bladder of the sheep and exhibited similar timed drug release as *in vitro* device testing (Fig. 4 and Fig. S4). After release from the device, the drug surrogate filled the entirety of the bladder as seen by X‐ray. The device was retained within the bladder awaiting evacuation through urination.

### Docetaxel Delivery into the Bladder Wall Showed no Improvement with up to 4 h Duration of Gemcitabine Exposure

**Fig. 5:**
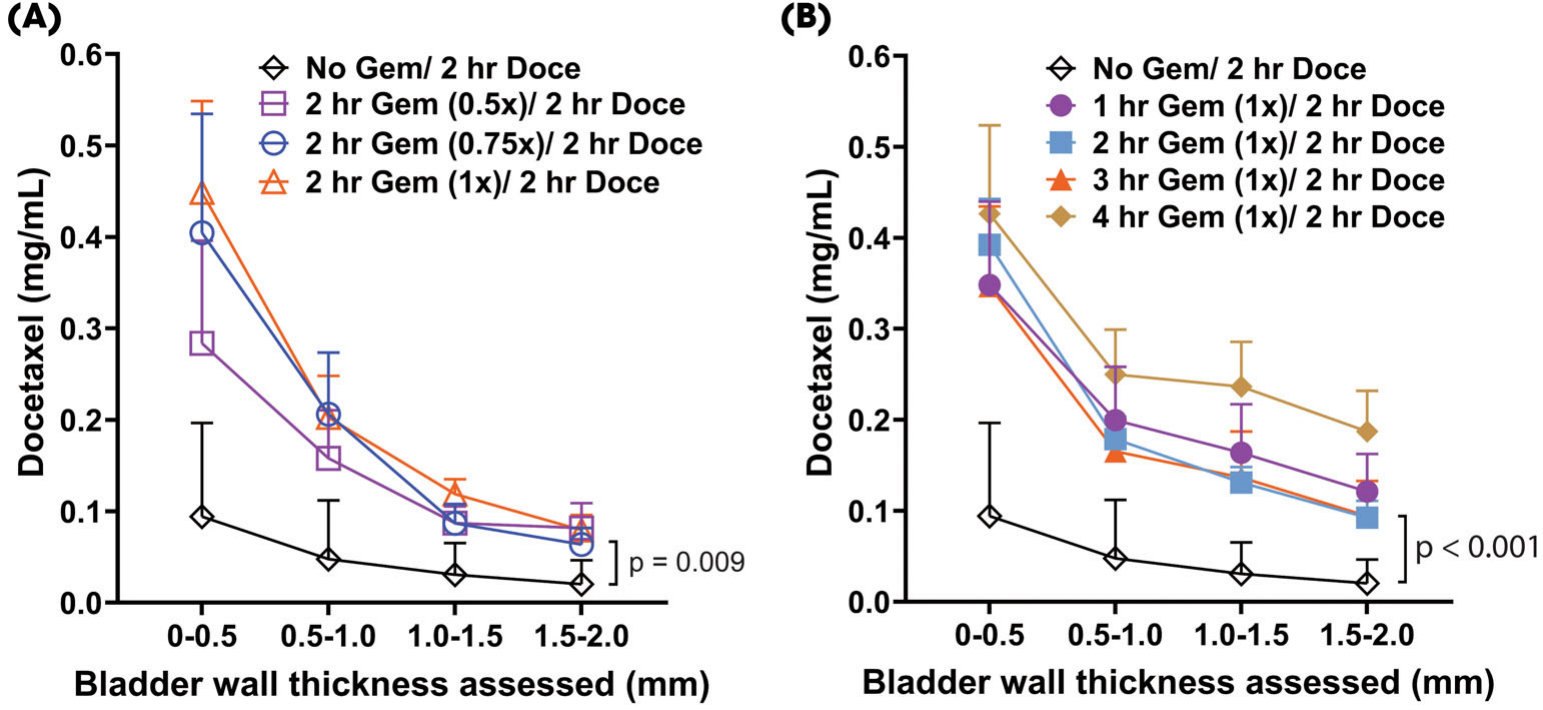
The impact of time and concentration of gemcitabine pre‐wash on docetaxel uptake in the bladder. (**A**) Docetaxel concentration as a function of gemcitabine concentrations (0×, 0.5×, 0.75×, and 1×). (**B**) Docetaxel concentration as a function of the duration of gemcitabine pre‐wash (0, 1, 2, 3, and 4 h). *P* values were determined by two‐way anova with multiple comparisons. *P* values were included where there was a statistical difference between groups.

Given the control over drug release from the DRIFT device, we sought to determine the influence of timing and concentration of gemcitabine exposure on docetaxel uptake into the bladder wall. Escalating gemcitabine concentrations (0.5×, 0.75×, 1×) led to higher docetaxel levels across all bladder wall depths compared to no gemcitabine pretreatment (Fig. 5A). At the 0–0.5 mm depth, docetaxel concentrations peaked at ~0.45 mg/mL with 1× gemcitabine vs ~0.08 mg/mL in controls, with the enhanced penetration persisting through 2.0 mm and reaching significance at the deepest layer (*P* = 0.009). Further, extending gemcitabine dwell time from 1 to 4 h (all at 1× concentration) also improved docetaxel delivery, with the 4‐h group achieving the highest levels at all depths (Fig. 5B). At 1.5–2.0 mm, the 4‐h group reached ~0.20 mg/mL compared to ~0.01 mg/mL in controls, demonstrating a significant improvement in deep tissue penetration (*P* < 0.001). There was limited docetaxel penetration without gemcitabine.

## Discussion

This study introduces a novel intravesical drug delivery device designed to enable controlled, delayed delivery of docetaxel directly into the bladder for the treatment of NMIBC. Our key findings indicate that the DRIFT device reliably maintains drug containment for up to 120‐min gemcitabine pre‐treatment period before releasing docetaxel. The buoyant design and structural flexibility of the DRIFT device effectively prevent urethral obstruction, allowing for continuous urine flow during treatment. In addition, the device can be removed by the patient after deployment of the docetaxel.

Previous studies in NMIBC treatment have highlighted the clinical benefit of Gem/Doce therapy, particularly in enhancing drug penetration into bladder tissues [3, 4, 6, 7, 8, 9, 11, 12, 13, 18]. Our findings reinforce the importance of a timed‐release mechanism, aligning with earlier reports that demonstrate improved tissue concentrations of docetaxel following exposure to gemcitabine [19]. However, earlier drug delivery methods lacked precise temporal control, often requiring prolonged clinic visits and specialised handling, limitations that our device effectively addresses [20]. The aetiology of improved docetaxel permeability by increasing concentration and duration of gemcitabine remains unclear, and this is presumed to be from mucosal disruption. Additional studies may allow for further optimisations.

A notable limitation of this study is the absence of *in vivo* biocompatibility and safety assessments; thus, conclusions regarding clinical tolerance and potential adverse reactions remain preliminary. Additionally, the experimental setup used simulated bladder environments that may not fully replicate the complex physiological conditions encountered clinically. These early‐stage studies are an important step in the development process, and subsequent *in vivo* validation will be critical to further establish the DRIFT device's safety and therapeutic potential in clinical settings. Furthermore, *in vivo* testing will assess for possible oncological benefits from optimisation of drug exposure. It will be important to develop the device such that the balloon may preferentially release when the bladder is empty, to optimise concentration. We aim to allow for atraumatic insertion and subsequent removal from the bladder via pulling the suture, similar to removal of a ureteric stent. Our next step will be to proceed with *in vivo* testing.

Despite these limitations, our approach offers substantial strengths. The DRIFT device is designed for integration with standard clinical equipment, allowing use by general healthcare personnel without specialised urological training. The flexibility in controlling drug‐release kinetics and the ability to customise device dimensions further enhance its potential applicability across diverse clinical scenarios. Clinically, this technology has the potential to significantly streamline NMIBC treatment by enabling the efficient, sequential administration of Gem/Doce in a single outpatient visit, potentially increasing patient compliance and improving therapeutic outcomes.

The next steps involve rigorous cytotoxicity and biocompatibility studies, as well as testing in large animal models to confirm the practical use, retention, and safety of the device under realistic conditions. Overall, this innovative intravesical drug delivery device represents a promising advancement, with significant potential for enhancing treatment protocols not only for NMIBC but also for other bladder conditions such as recurrent UTIs and interstitial cystitis.

## Disclosure of Interests

Ashley C. Rhodes, Emily Witt, Vignesh T. Packiam, and James D. Byrne have filed a provisional patent related to the DRIFT device. Vignesh T. Packiam additionally serves as a consultant and/or speaker for Valar Labs, Veracyte, Photocure, Ferring, and Johnson & Johnson. Michael A. O’Donnell has consulting relationships with Abbott, Photocure, Fidia, Merck, Theralase, and Urogen. All other authors declare no conflicts of interest.

## Supporting information


**Fig. S1.** Serial images depict device filling via a pre‐inserted 26‐G needle connected to a ~1.19 mm (3/64″) polypropylene tube through the silicone end cap.
**Fig. S2.** (**A**) Top and (**B**) side images depicting the DRIFT device floating in water.
**Fig. S3.** Inflation pressure was evaluated for the DRIFT device.
**Fig. S4.** Serial images depicting the dissolution of the assembled device.
