# Supplementary material for: Drug‐releasing intravesical floating technology for sequential gemcitabine and docetaxel in non‐muscle‐invasive bladder cancer
Source: BJU Int. 2025 Nov 3;137(2):360–7. doi: 10.1111/bju.70060 (PMC12789840; doi:10.1111/bju.70060)
Supplement: Supplementary file 1 — Fig. S1. Serial images depict device filling via a pre‐inserted 26‐G needle connected to a ~1.19 mm (3/64″) polypropylene tube through the silicone end cap. Fig. S2. (A) Top and (B) side images depicting the DRIFT device floating in water. Fig. S3. Inflation pressure was evaluated for the DRIFT device. Fig. S4. Serial images depicting the dissolution of the assembled device. [file BJU-137-360-s001.docx]

**Supplemental Information:**

**
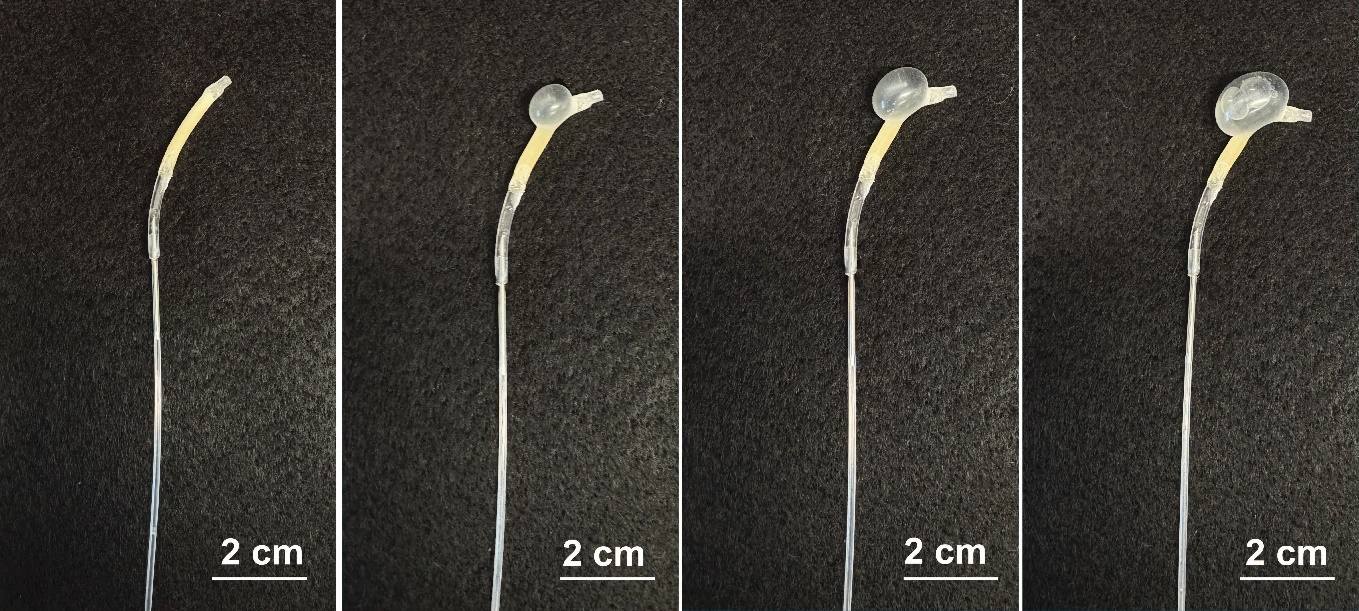
**

**Fig. S1.** Serial images depict device filling via a pre-inserted 26G needle connected to a 3/64” polypropylene tube through the silicone end cap. For DRIFT device insertion and filling, the needle and tube are preloaded and remain attached as the device is expelled from the Foley catheter. After the device is filled, the needle and tube are removed from the catheter.


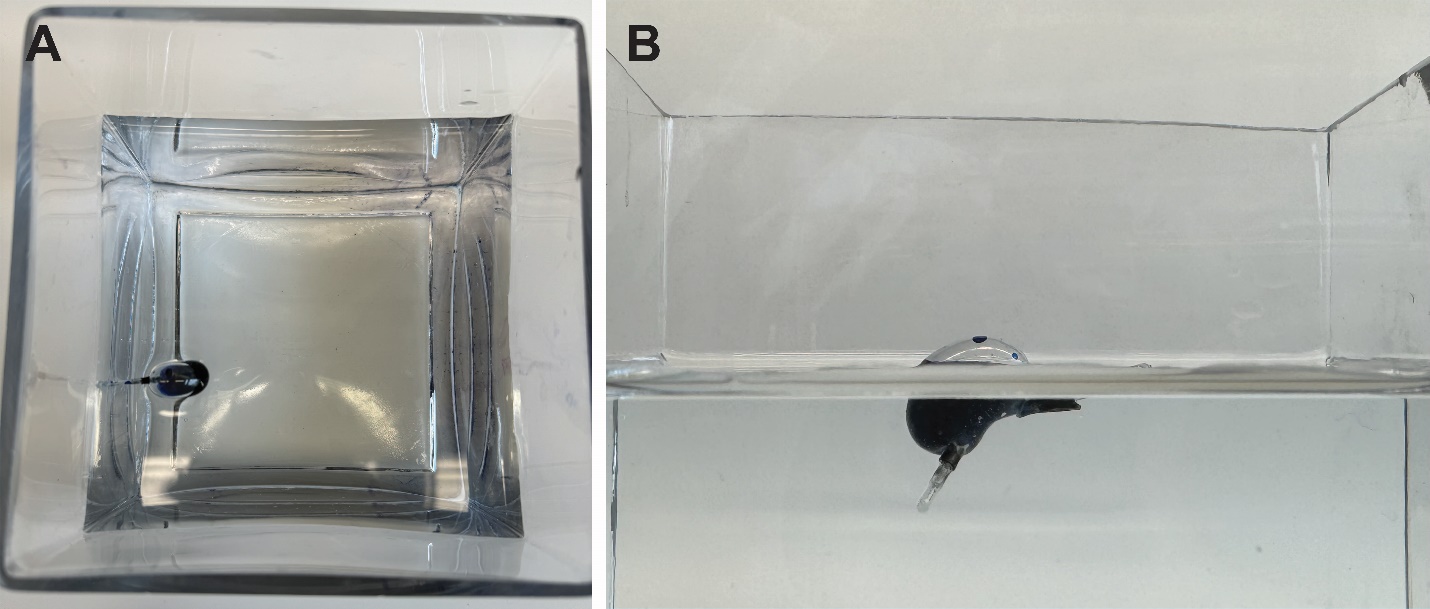


**Fig. S2.** (A) Top and (B) side images depicting the DRIFT device floating in water. Methylene blue (0.5% w/v) was used as a drug substitute.


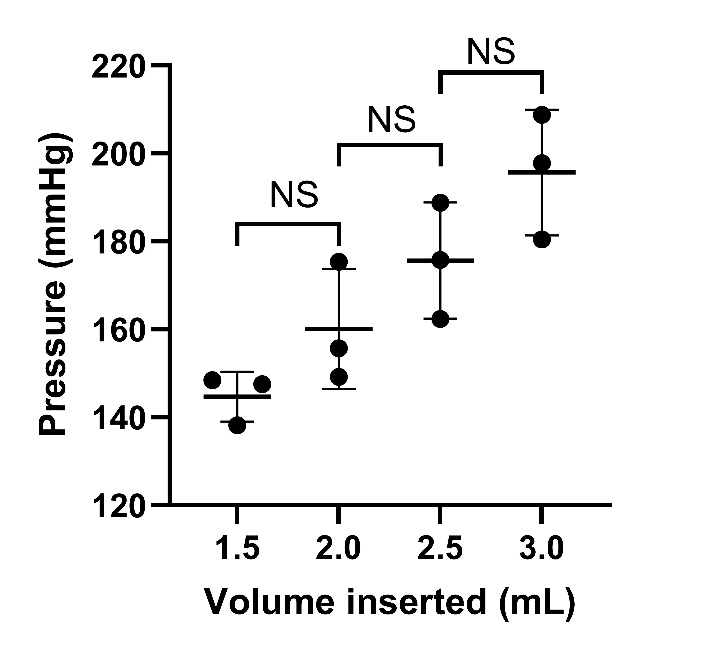


**Fig. S3.** Inflation pressure was evaluated for the DRIFT device. Pressure in a 22-gauge needle as a function of volume injected into each device. P values were determined by one-way ANOVA with multiple comparisons. NS – not significant.

**
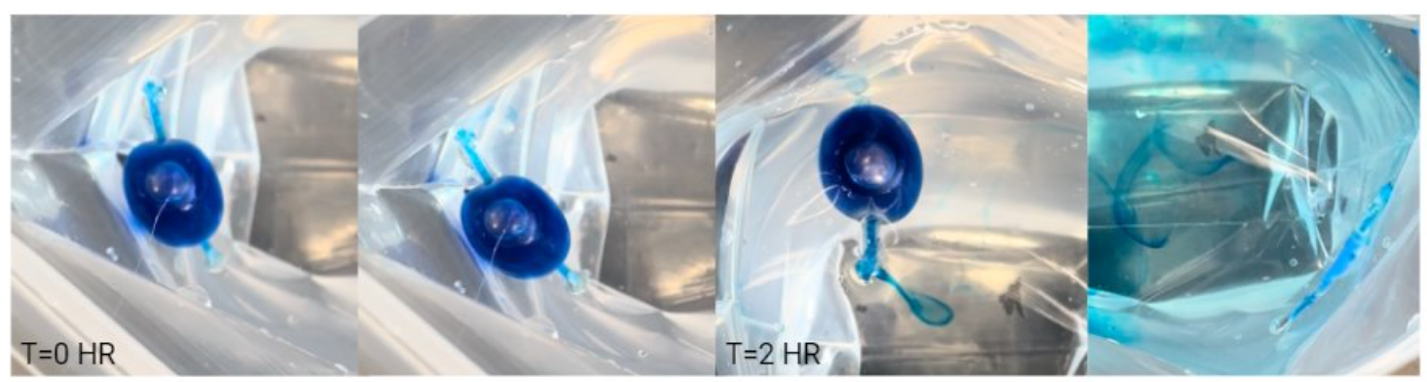
**

**Fig. S4.** Serial images depicting the dissolution of the assembled device. Methylene blue (0.5% w/v) was used as a drug substitute.
